# Association of Symptoms and Viral Culture Positivity for SARS‐CoV‐2—Tennessee, April–July 2020

**DOI:** 10.1111/irv.13318

**Published:** 2024-06-21

**Authors:** Jessica E. Biddle, Gaston Bonenfant, Carlos G. Grijalva, Yuwei Zhu, Natasha B. Halasa, James D. Chappell, Alexandra Mellis, Carrie Reed, H. Keipp Talbot, Bin Zhou, Melissa A. Rolfes

**Affiliations:** ^1^ Influenza Division Centers for Disease Control and Prevention Atlanta Georgia USA; ^2^ Department of Health Policy Vanderbilt University Medical Center Nashville Tennessee USA; ^3^ Department of Biomedical Informatics Vanderbilt University Medical Center Nashville Tennessee USA; ^4^ Department of Biostatistics Vanderbilt University Medical Center Nashville Tennessee USA; ^5^ Department of Pediatrics Vanderbilt University Medical Center Nashville Tennessee USA; ^6^ Department of Medicine Vanderbilt University Medical Center Nashville Tennessee USA

**Keywords:** culture positivity, SARS‐CoV‐2, symptoms, viral culture

## Abstract

**Background:**

Understanding how symptoms are associated with SARS‐CoV‐2 culture positivity is important for isolation and transmission control guidelines.

**Methods:**

Individuals acutely infected with SARS‐CoV‐2 in Tennessee and their household contacts were recruited into a prospective study. All participants self‐collected nasal swabs daily for 14 days and completed symptom diaries from the day of illness onset through day 14 postenrollment. Nasal specimens were tested for SARS‐CoV‐2 using RT‐qPCR. Positive specimens with cycle threshold values < 40 were sent to the Centers for Disease Control and Prevention (CDC) for viral culture. First, we modeled the association between symptoms and the risk of culture positivity using an age‐adjusted generalized additive model (GAM) accounting for repeated measurements within participants and a symptom‐day spline. Next, we investigated how timing of symptom resolution was associated with the timing of culture resolution.

**Results:**

In a GAM restricted to follow‐up days after symptoms began, the odds of a specimen being culture positive was significantly increased on days when wheezing, loss of taste or smell, runny nose, nasal congestion, sore throat, fever, or any symptom were reported. For all symptoms except sore throat, it was more common for participants to have culture resolution before symptom resolution than for culture to resolve after or on the same day as symptom resolution.

**Conclusions:**

Overall, symptomatic individuals were more likely to be SARS‐CoV‐2 viral culture positive. For most symptoms, culture positivity was more likely to end before symptoms resolved. However, a proportion of individuals remained culture positive after symptom resolved, across all symptoms.

## 1 Introduction

Individuals with coronavirus disease 2019 (COVID‐19) can have a wide range of symptoms [1] that typically start 2–14 days after exposure to severe acute respiratory syndrome coronavirus‐2 (SARS‐CoV‐2) and persist for days to weeks in some instances [2]. Current Centers for Disease Control and Prevention (CDC) recommendations suggest that individuals testing positive for SARS‐CoV‐2 infection should isolate from others to reduce transmission [3]. For asymptomatic or mildly symptomatic individuals, self‐isolation can be discontinued after 5 days if symptoms are improving and they are afebrile for 24 h without fever‐reducing medication based on the assumption that they are most likely infectious during the first 5 days. [3]. However, not all SARS‐CoV‐2 infections result in fever and few studies have examined the longitudinal natural history of infection dynamics in conjunction with symptoms.

Overall, studies looking at the relationship between SARS‐CoV‐2 viral culture positivity and SARS‐CoV‐2 transmission are scarce. However, within a household setting, we have previously shown that there may be an increased risk of secondary infection among household contacts when primary cases are culture positive for longer than 5 days after illness onset [4]. Viral culture positivity may be a proxy for infectiousness, underscoring the importance of understanding how symptoms are associated with viral culture positivity. Furthermore, understanding how individual symptoms are associated with infections caused by SARS‐CoV‐2 virus can provide valuable knowledge for future public health recommendations for isolation and transmission control.

Therefore, we aimed to evaluate how symptoms were associated with wild‐type SARS‐CoV‐2 culturable (i.e., possibly infectious) virus using daily respiratory specimen collection and symptom reporting. We also aimed to describe the risk of having wild‐type SARS‐CoV‐2 culturable virus relative to the presence of symptoms during follow‐up and explored the relationship between the timing of symptom resolution and culture positivity resolution.

## 2 Methods

### 2.1 Study Design and Sample Collection

We conducted a case‐ascertained household transmission study of SARS‐CoV‐2 in Nashville, Tennessee from April to September 2021 [5]. We used only households recruited from April to July 2020 for the present analyses due to availability of viral culture results. We recruited individuals acutely infected with SARS‐CoV‐2 that were the first identified in the household (index case) and their household contacts into the study if the index case had illness onset within 7 days of enrollment and there was at least one non‐ill household contact at the time of the index case disease onset. Informed consent was obtained from the index cases and their household contacts prior to study activities. The protocol was reviewed and approved by the institutional review board at Vanderbilt University Medical Center and the CDC determined study activities were conducted consistent with applicable federal law and CDC policy (See 45 C.F.R. part 46; 21 C.F.R. part 56).

All participants (index cases and household contacts) self‐reported demographic and household characteristics at enrollment and were instructed to self‐collect nasal swabs and record information on symptoms daily for the following 14 days. Nasal swab specimens were tested for SARS‐CoV‐2 using reverse transcription quantitative polymerase chain reaction (RT‐qPCR) [6]. On daily symptom diaries, participants reported presence or absence of fever, cough, sore throat, runny nose, nasal congestion, fatigue, wheezing, shortness of breath, headache, and loss of taste and smell. Participants aged ≥ 18 years that reported at least one of these symptoms on a given day also completed the validated Influenza Intensity and Impact Questionnaire (FluiiQ) [7] daily starting on the day of symptom onset through the end of follow‐up. The FluiiQ utilizes a 4‐point Likert scale to express severity of a specific symptom on a given day: 0 (*the symptom was not experienced*), 1 (*symptom was mild*), 2 (*moderate*), to 3 (*severe*) [7]. Participants are asked to complete the questionnaire right before going to bed each night and instructed to rate each symptom, thinking about when they felt the worst over the past 24 h. For this analysis, we focused on the daily FluiiQ results for fever, cough, nasal congestion, sore throat, headache, and fatigue. Of note, the FluiiQ is validated for influenza virus infection and was used in an exploratory way for this analysis.

### 2.2 Viral Cultures

Nasal swab specimens that were RT‐qPCR positive with cycle threshold (CT) value < 40 for any SARS‐CoV‐2 viral target (N1, N2) were sent to CDC (Atlanta, GA) for viral culture. Specimens were inoculated onto Vero E6‐TMRPSS2 cells and cytopathic effects were recorded between 3 and 7 days after inoculation, as previously described [8]. Results were reported as culture‐positive or culture‐negative, and RT‐qPCR was used to confirm SARS‐CoV‐2 in presumptive positive cultures.

### 2.3 Statistical Analysis

We included participants in analysis if they had ≥ 2 RT‐qPCR (PCR) positive swabs during follow‐up with at least one of those swabs meeting the criteria for viral culture. We categorized study follow‐up days as PCR positive/culture positive, PCR positive/culture negative, or PCR negative. We assumed that specimens with an invalid or inconclusive PCR result were PCR negative. Additionally, we assumed that a specimen that was PCR positive but with culture status unknown (due to an issue during the attempted culture assay) was culture negative.

We estimated the odds of being culture positive given the presence or absence of any symptom (defined as having reported at least one symptom) on a given day using a logistic regression. A generalized additive model (GAM) with a binomial distribution using a logit link function was used to account for repeated measurements within participants, adjusted for age as a continuous variable, with a thin‐plate spline to account for the nonlinear relationship between symptom‐day and culture status. PCR negative days were classified as culture negative. We restricted the model to include only participants that ever‐reported symptoms and only their follow‐up days after symptoms began. Using the same parameters, we repeated the age‐adjusted GAMs for each specific symptom. We restricted each symptom‐specific model to include only participants that ever‐reported the specific symptom and only their follow‐up days after that specific symptom began. We conducted two sensitivity analyses. First, we excluded specimens that were PCR invalid or inconclusive and specimens that were PCR positive with an unknown culture result. Second, all analyses were repeated among only follow‐up days of ever‐symptomatic participants when the participant had positive PCR results. We also conducted an additional crude analysis to estimate the odds of being culture positive given the presence or absence of any symptom stratified by days from symptom onset.

We also examined symptom severity from the FluiiQ responses to further examine the relationship between symptoms severity and SARS‐CoV‐2 viral culture positivity. We estimated the risk of having a culture positive result on a given day of follow‐up after symptoms began, by self‐reported symptom severity (mild, moderate, severe) compared to not reporting the given symptom using age‐adjusted GAMs for each symptom, accounting for repeated measurement within participants and with a spline to account for symptom‐day.

Next, we investigated how timing of symptom resolution was associated with the timing of culture positive resolution/conversion to negative. For each participant, we defined symptom resolution as the day after the latest reported symptom‐day and culture resolution as the day after the last positive viral culture was observed in follow‐up. We accounted for possible intermittent asymptomatic or culture negative days by taking the last observed symptom or culture positive day regardless of an asymptomatic or culture negative day occurred before that. Among individuals that were ever‐culture positive we examined whether culture resolution occurred before, after, or on the same day as symptom resolution. If either culture resolution or symptom resolution occurred during follow‐up, we assumed that the observed order of occurrence was reflective of the actual temporal pattern of events. If neither culture resolution nor symptom resolution occurred during the follow‐up period, we classified both as unknown.

Analyses were conducted using R (Version 4.0.3) [9] and RStudio (Version 1.3.1056) [10]. Generalized additive models were conducted using the mgcv package [11].

## 3 Results

One hundred and eleven participants (43 households) were enrolled from April to July 2020. Thirty‐four participants were never PCR positive during follow‐up and were excluded. An additional 13 participants were excluded for not meeting the inclusion criteria of 2 or more PCR positive swabs during follow‐up with at least one of those swabs meeting the criteria for viral culture assay. After exclusions, there were 64 participants (38 index cases and 26 household contacts) included in analysis; 61% female, 72% aged 18–49 years, 42% were Hispanic, 50% were non‐Hispanic white, nearly all (94%) reported at least one symptom on at least 1 day during follow‐up, and 86% had at least 1 positive viral culture during follow‐up (Table 1). The most common symptoms reported were fatigue (81%), cough (67%), loss of taste or smell (67%), and nasal congestion (66%). Fever, runny nose, and headache were reported in over half the participants (Table 1). Fifty‐two (81%) of participants reported using medication to reduce fever and/or for pain at least one day during follow‐up (Table 1).

**TABLE 1:** Characteristics of included SARS‐CoV‐2 positive study participants—Nashville, TN, 2020.

		*n*	%
Overall		64	
Participant type	Index	38	59.4
	Household contact	26	40.6
Sex	Female	39	60.9
	Male	25	39.1
Age group (years)	< 5	1	1.6
	5–11	4	6.2
	12–17	4	6.2
	18–29	23	35.9
	30–49	23	35.9
	50–64	7	10.9
	65+	2	3.1
Race/ethnicity	Hispanic	27	42.2
	Non‐White, non‐Hispanic	5	7.8
	White, non‐Hispanic	32	50.0
Symptom ever reported	Any symptom	60	93.8
	Fatigue	52	81.2
	Cough	43	67.2
	Loss of taste or smell	43	67.2
	Nasal congestion	42	65.6
	Fever	37	57.8
	Runny nose	35	54.7
	Headache	34	53.1
	Shortness of breath	30	46.9
	Chest tightness/pain	27	42.2
	Sore throat	25	39.1
	Wheeze	16	25.0
Culture status	Ever culture positive	55	85.9
	Never culture positive	9	14.1
Fever/pain medication use	Reported at least once	52	81.2

Overall, there were 844 specimens collected from the 64 participants during follow‐up. Of these, 19 specimens had invalid/inconclusive PCR results and were assumed PCR negative. Thirty‐one specimens were PCR positive but with unknown culture status due to an issue during the attempted culture assay and were assumed culture negative. Fifty‐five participants (86% of 64) were culture positive for at least 1 day and a median of 3.0 days (IQR 2–5 days; range: 1–14 days) from first culture positive specimen to last culture specimen observed during follow‐up. Of the 60 symptomatic participants, 52 (87%) were culture positive for at least 1 day during follow‐up. There was a total of 748 specimens with PCR results among symptomatic individuals when we restricted data to the period after symptoms began. Among the four participants that were asymptomatic throughout follow‐up, 3 (75%) were culture positive for 1, 2, or 4 days of follow‐up.

**FIGURE 1:**
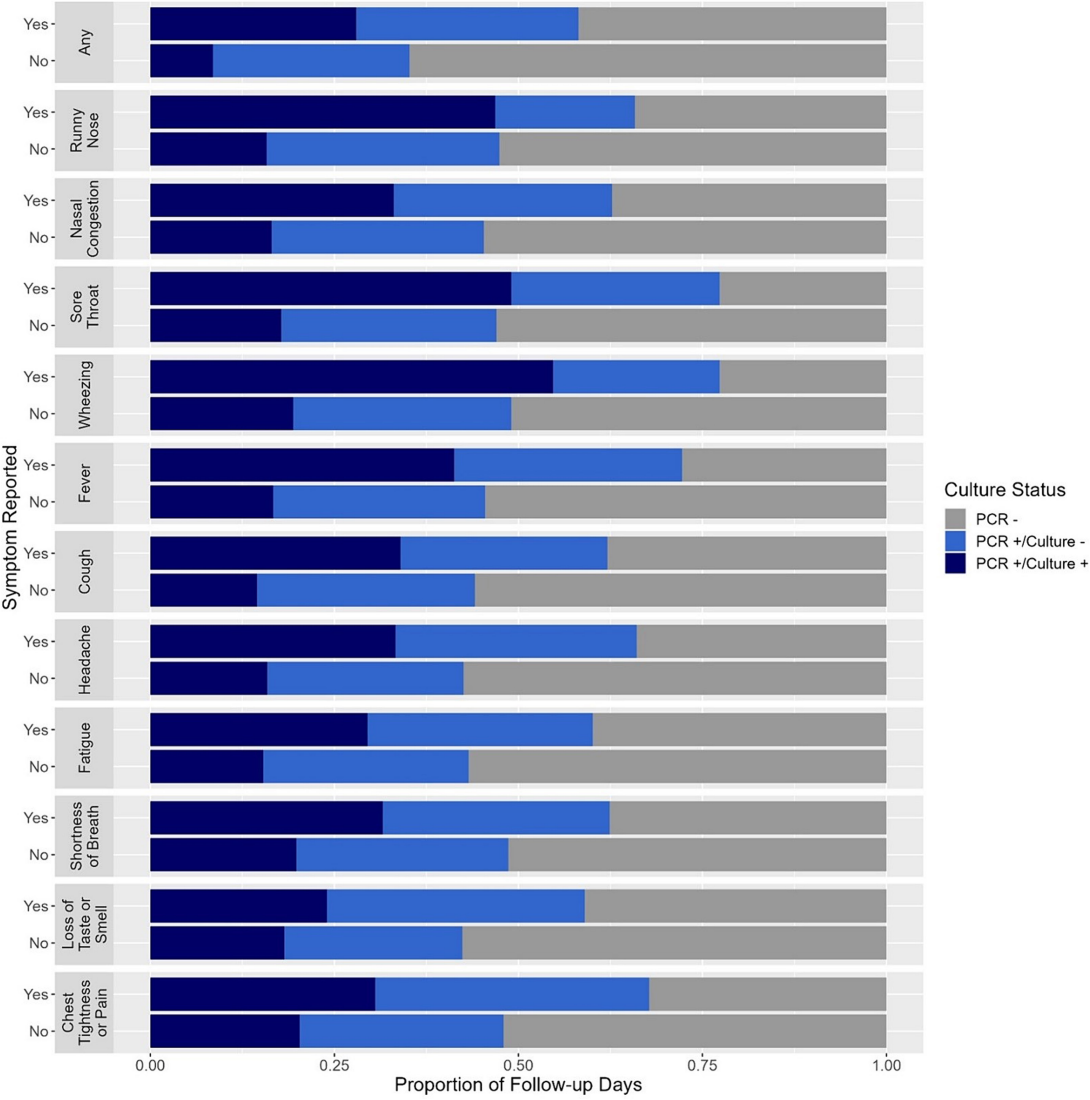
Proportion of SARS‐CoV‐2 PCR positive/culture positive, PCR positive/culture negative, and PCR negative specimens among symptomatic individuals during follow‐up, by symptom—Nashville, TN, 2020. This includes all follow‐up days. For any symptom and each individual symptom, the probability of having a culture positive specimen on days when symptom was present is increased for each symptom compared to days when symptom absent.

**FIGURE 2:**
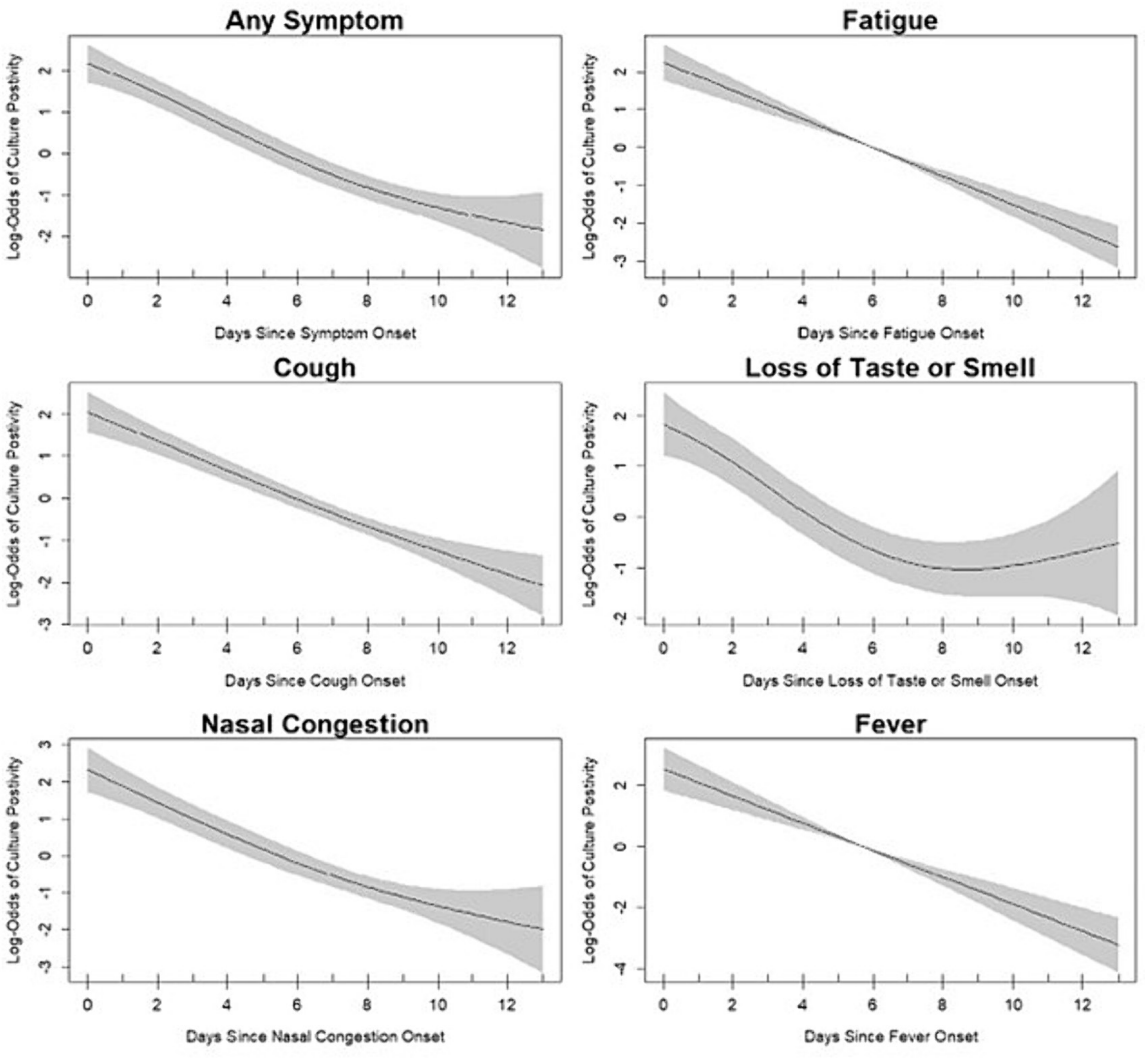
Odds of culture positivity by symptom‐day—Nashville, TN, 2020. Each subplot illustrates the nonlinear relationship between symptom‐day and culture status using the GAM described above for each symptom listed.

Overall, on follow‐up days when participants reported having any symptom (median of 3.0 symptoms; IQR 2–6 symptoms), 28% of specimens was culture positive compared with 9% on days when no symptoms were reported (Figure 1). Furthermore, for every individual symptom, the probability of having a culture positive specimen on days when the symptom was present was increased for each symptom compared to days when symptom absent (Figure 1). In a GAM restricted to follow‐up days after a symptom began and controlling for age and symptom‐day, the odds of a specimen being culture positive was significantly increased on days when wheezing (odds ratio [OR]: 11.57; 95% confidence internal [CI]: 3.81–35.14), loss of taste or smell (OR: 3.43; CI: 1.43–8.23), runny nose (OR: 3.21; CI: 1.79–5.77), nasal congestion (OR: 3.07; CI: 1.63–5.77), sore throat (OR: 2.70; CI: 1.32–5.32), fever (OR: 1.94; CI: 1.05–3.59), and any symptom (OR: 2.03; CI: 1.04–3.96) were reported (Table 2). For a subset of symptoms, we visualized the results of the GAM fitted to the data, specifically focusing on symptom‐day (Figure 2). When we excluded PCR invalid/inconclusive and PCR positive with an unknown culture specimen, these days all remained significant except any symptom and fever (Table S1). When we restricted to known PCR positive days, these symptoms all remained significant again except for any symptom and fever (Table S2). When we stratified by days since symptom onset, we found that on days 0–4 from symptom onset participants were culture positive on 49% of symptomatic days and on days 5+ from symptom onset participants were culture positive on 9% of symptomatic days (Table S3).

**TABLE 2:** Odds of SARS‐CoV‐2 culture positivity among symptomatic individuals, by symptom—Nashville, TN, 2020.

	Frequencies^a^		Odds of culture positivity on days with symptom compared to days without symptom^a^			
Symptom	Total person‐days	Culture positive days/days with symptom	Crude OR	Model‐based adjusted OR^b^	Model‐based adjusted 95% CI^b^	Model‐based *p*‐value^b^
Any symptom ^c^	748	154/550 (28%)	4.26	2.03	[1.04, 3.96]	< 0.05
Wheezing	171	29/53 (55%)	5.87	11.57	[3.81, 35.14]	< 0.0001
Loss of taste or smell	480	65/271 (24%)	6.27	3.43	[1.43, 8.23]	< 0.01
Runny nose	420	74/158 (47%)	4.09	3.21	[1.79, 5.77]	< 0.0001
Nasal congestion	520	87/263 (33%)	5.00	3.07	[1.63, 5.77]	< 0.0001
Sore throat	299	52/106 (49%)	3.94	2.70	[1.32, 5.32]	< 0.01
Fever	422	64/155 (41%)	3.67	1.94	[1.05, 3.59]	< 0.05
Headache	350	57/171 (33%)	3.98	1.98	[0.94, 4.15]	0.07
Shortness of breath	327	42/133 (32%)	3.40	1.96	[0.97, 3.93]	0.06
Cough	541	104/306 (34%)	2.58	1.63	[0.98, 2.71]	0.06
Chest tightness or pain	302	37/121 (31%)	2.91	1.45	[0.67, 3.15]	0.343
Fatigue	639	108/366 (30%)	2.18	1.11	[0.67, 1.84]	0.682

^a^ Restricted to follow‐up days after given symptom began. Each row is a separate model.

^b^ An age‐adjusted generalized additive model (GAM) with a binomial distribution using a logit link function was used to account for repeated measurements within participants, with a thin‐plate spline to account for the nonlinear relationship between symptom‐day and culture status.

^c^ Any symptom is defined as having reported at least one symptom.

Among adult participants who completed the FluiiQ, the odds of being culture positive on that day increased with increasing reported severity of nasal congestion, sore throat, fever, and fatigue (Table S4). For example, among the 33 adult participants who reported fever, the specimen was culture positive on 73% of days in which participants reported their fever as “severe” compared with only 11% of days in which participants were afebrile (Figure S1).

Of the 51 symptomatic individuals that were culture positive at least 1 day during follow‐up, 47 (92%) had culture resolution observed during the 14‐day follow‐up. Of the 51, 86% had culture resolution before all symptoms had resolved, 8% remained culture positive until after all symptoms resolved, 4% had symptom and culture resolution on the same day, and 1 continued to have symptoms and was culture positive on the last follow‐up day (Table 3). The timing of resolution varied by symptom, but for most symptoms, it was more common for participants to have culture resolution before symptom resolution than for culture to resolve after or on the same day as symptom resolution (Table 3).

**TABLE 3:** Timing of culture resolution compared to symptom resolution among individuals that were ever culture positive for SARS‐CoV‐2, by symptom—Nashville, TN, 2020.

				Timing of culture resolution							
	Overall symptomatic individuals	Days to symptom resolution		Before symptom resolution		After symptom resolution		Same day as symptom resolution		Unknown^b^	
Symptom	*n*	Median	IQR	*n*	%	*n*	%	*n*	%	*n*	%
All symptoms ^a^	51	10	7–13	44	86.3%	4	7.8%	2	3.9%	1	2.0%
Fatigue	44	7.5	3–12.25	31	70.5%	8	18.2%	4	9.1%	1	2.3%
Headache	27	6	2–9.5	19	70.4%	8	29.6%	0	0.0%	0	0.0%
Chest tightness/pain	21	4	2–8	15	71.4%	5	23.8%	1	4.8%	0	0.0%
Cough	39	6	2.5–12	24	61.5%	13	33.3%	1	2.6%	1	2.6%
Fever	31	4	1–8.5	18	58.1%	7	22.6%	5	16.1%	1	3.2%
Runny nose	31	3	1–10	15	48.4%	10	32.3%	6	19.4%	0	0.0%
Sore throat	24	2	0–7.5	10	41.7%	11	45.8%	3	12.5%	0	0.0%
Shortness of breath	22	6	3–9.75	15	68.2%	5	22.7%	2	9.1%	0	0.0%
Nasal congestion	36	7	2.75–12	24	66.7%	7	19.4%	5	13.9%	0	0.0%
Loss of taste or smell	29	7	4–9	23	79.3%	3	10.3%	3	10.3%	0	0.0%
Wheezing	13	4	0–7	6	46.2%	5	38.5%	2	15.4%	0	0.0%

^a^ For all symptoms, resolution occurred when all symptoms had resolved.

^b^ Four individuals were still culture positive at the end of follow‐up; one was still symptomatic at end of follow‐up therefore unknown; the remaining three had symptoms resolve before end of follow‐up.

## 4 Discussion

In our 2020 household transmission study of wild‐type SARS‐CoV‐2, we found that having symptoms of acute respiratory infection, such as fever, runny nose, nasal congestion, sore throat, loss of taste/smell, and wheezing, significantly predicted being culture positive on that day. Currently, mildly symptomatic individuals positive for SARS‐CoV‐2 are encouraged to self‐isolate for the first 5 days after symptom onset. If symptoms are improving after 5 days, they can end isolation but wear a mask in public and at home around others once they are afebrile for 24 h without fever‐reducing medication. For individuals positive for SARS‐CoV‐2 with moderate illness, self‐isolation is recommended through day 10 of symptoms onset and those with severe illness should also self‐isolate through day 10 and consult with their doctor before ending isolation [3]. These time‐based isolation recommendations are meant to reduce onward transmission. Using data from a study conducted before the use of COVID‐19 vaccines that included daily respiratory specimen collection and symptom reporting in individuals with wild‐type SARS‐CoV‐2 infection, we evaluated how self‐reported symptoms were associated with culturable virus, and therefore infectiousness, to gain knowledge for future public health recommendations for isolation and control by evaluating the premise of symptom‐based isolation guidance.

We found when a person is exposed to COVID‐19, but PCR status is unknown, the development of wheezing, runny nose, sore throat, nasal congestion, fever, loss of taste or smell, and shortness of breath were all predictive of culture positivity and that day since symptom onset plays a role in the probability of culture positivity. We also found that culture positivity was more likely to end before a symptom was resolved than after; however, the proportion of people who remained culture positive after the symptom resolved varied from 18% to 45% depending on the symptom. Future public health recommendations could consider resolution of other symptoms in addition to fever, for guidance on ending isolation.

While previous studies have demonstrated a relationship between viral culture positivity and SARS‐CoV‐2 transmission, culture status at the time of infection and during illness is not available [4]. Our findings affirm that certain symptoms can provide information for infectiousness and could be used to guide isolation to mitigate transmission. Other studies have shown that viral RNA load is correlated with symptom severity, specifically, higher estimated load in patients with greater symptom severity [12]. We observed a similar association in the present study with culture positivity being positively associated with the presence of symptoms and increased symptom severity among adults. However, evidence on the relationship between symptoms and transmission is mixed and is likely dependent on age and prior history of SARS‐CoV‐2 infection and COVID‐19 vaccination. One household study found that index case symptom status did not affect cumulative infection rate [13]. Our study was not sufficiently powered to address this question and additional research is warranted to fully understand the relationships between SARS‐CoV‐2 viral load, culture positivity, symptoms, day of illness, and transmission risk.

There are several limitations to our study and analysis. First, the study took place early in the COVID‐19 pandemic when individuals did not have any preexisting SARS‐CoV‐2 immunity including vaccination or prior infection and before the emergence of the first variant of concern. Thus, our findings may not be generalizable to the current state of COVID‐19. Specifically, previous studies have shown that COVID‐19 symptoms have evolved with new SARS‐CoV‐2 variants [14, 15]. However, understanding data from early in the pandemic remains relevant to serve as a resource for ongoing public health recommendations and future pandemic preparedness. Our study had a small number of participants 65 and older; therefore, the results may not be generalizable to the elderly. Additionally, our study included mostly symptomatic individuals, and future work could explore culture positivity in depth among a population with more asymptomatic individuals. Furthermore, we did not observe the end of symptoms or the end of culture positivity for all individuals due to the duration of follow‐up. Considering the long duration of COVID‐19 symptoms in some people, future work should sample individuals until the end of symptoms and culture positivity are observed. A limitation of the FluiiQ tool is the severity of symptoms are self‐reported and therefore subjective. Also, we note that the FluiiQ tool was not validated for children at the time of our study; thus, assessing symptom severity in children could be the focus of future work.

Additionally, we restricted our analysis to participants that had two or more PCR positive swabs during follow‐up which could potentially bias toward including individuals with more severe or longer duration of illness. Next, while our study focused on the binary distinction between culture positive and negative due to the data limitations at the time, we acknowledge that quantitative viral load measurements would provide a more nuanced understanding of infectiousness. Future work should include quantification of viral load to allow for a more precise assessment of the amount of infectious virus present in a sample. Finally, while SARS‐CoV‐2 isolation in cell culture demonstrates replication‐competent virus in a respiratory specimen and indicates the potential to initiate infection in susceptible contacts, the relationship between SARS‐CoV‐2 infectivity in vitro and transmission between hosts has not been precisely determined.

Overall, people with wild‐type SARS‐CoV‐2 infection were significantly more likely to be culture positive on days when they reported symptoms; specifically, wheezing, runny nose, sore throat, nasal congestion, fever, loss of taste and smell, and shortness of breath were significantly associated with being culture positive. Data from adults also suggest that when someone feels that their symptoms are particularly severe, they are more likely to be culture positive and therefore infectious to others. Finally, while culture positivity was more likely to conclude before symptom resolution, there was variability by symptom and a considerable proportion of individuals remained culture positive after symptoms resolved. These results may be helpful for public health professionals and decision‐makers evaluating when to end isolation while reducing onward transmission of SARS‐CoV‐2.

## Author Contributions

**Jessica E. Biddle:** conceptualization (lead), formal analysis (lead), methodology (equal), writing–original draft (lead), writing–review and editing (equal). **Gaston Bonenfant:** methodology (supporting), writing–review and editing (supporting). **Carlos G. Grijalva:** funding acquisition (equal), investigation (equal), methodology (equal), writing–review and editing (equal). **Yuwei Zhu:** investigation (equal), methodology (equal), writing–review and editing (supporting). **Natasha B. Halasa:** investigation (equal), methodology (equal), writing–review and editing (supporting). **James D. Chappell:** investigation (equal), methodology (equal), writing–review and editing (supporting). **Alexandra Mellis:** conceptualization (equal), formal analysis (supporting), methodology (equal), writing–review and editing (equal). **Carrie Reed:** conceptualization (equal), funding acquisition (equal), writing–review and editing (supporting). **H. Keipp Talbot:** funding acquisition (equal), investigation (equal), methodology (equal), writing–review and editing (equal). **Bin Zhou:** data curation (supporting), methodology (supporting), writing–review and editing (supporting). **Melissa A. Rolfes:** conceptualization (equal), formal analysis (supporting), funding acquisition (equal), methodology (equal), writing–review and editing (equal).

## Conflicts of Interest

CGG reports grants from Campbell Alliance/Syneos, the National Institutes of Health, the Food and Drug Administration, the Agency for Health Care Research and Quality, and Sanofi‐Pasteur and consultation fees from Merck.

### Peer Review

The peer review history for this article is available at https://www.webofscience.com/api/gateway/wos/peer‐review/10.1111/irv.13318.

## Supporting information


**Figure S1.** Proportion of SARS‐CoV‐2 culture positive and culture negative specimens among symptomatic adults, by symptom severity—Nashville, TN, 2020. Symptom severity data comes from the validated Influenza Intensity and Impact Questionnaire (FluiiQ), which was asked of symptomatic participants aged ≥ 18 years.

**Table S1.** Odds of SARS‐CoV‐2 culture positivity among symptomatic individuals, by symptom—Nashville, TN, 2020: sensitivity analysis 1—exclusion of invalid specimens.
**Table S2.** Odds of SARS‐CoV‐2 culture positivity among symptomatic individuals on PCR positive follow‐up days, by symptom—Nashville, TN, 2020: sensitivity analysis 2—exclusion of PCR negative results.
**Table S3.** Odds of SARS‐CoV‐2 culture positivity among symptomatic individuals, by symptom and days since symptom onset—Nashville, TN, 2020.
**Table S4.** Odds of SARS‐CoV‐2 culture positivity among symptomatic adults, by symptom severity—Nashville, TN, 2020.

## Data Availability

The data that support the findings of this study are available upon reasonable request.
