# Supplementary figures and images for: Association of Symptoms and Viral Culture Positivity for SARS‐CoV‐2—Tennessee, April–July 2020
Source: Influenza Other Respir Viruses. 2024 Jun 21;18(6):e13318. doi: 10.1111/irv.13318 (PMC11190945; doi:10.1111/irv.13318)

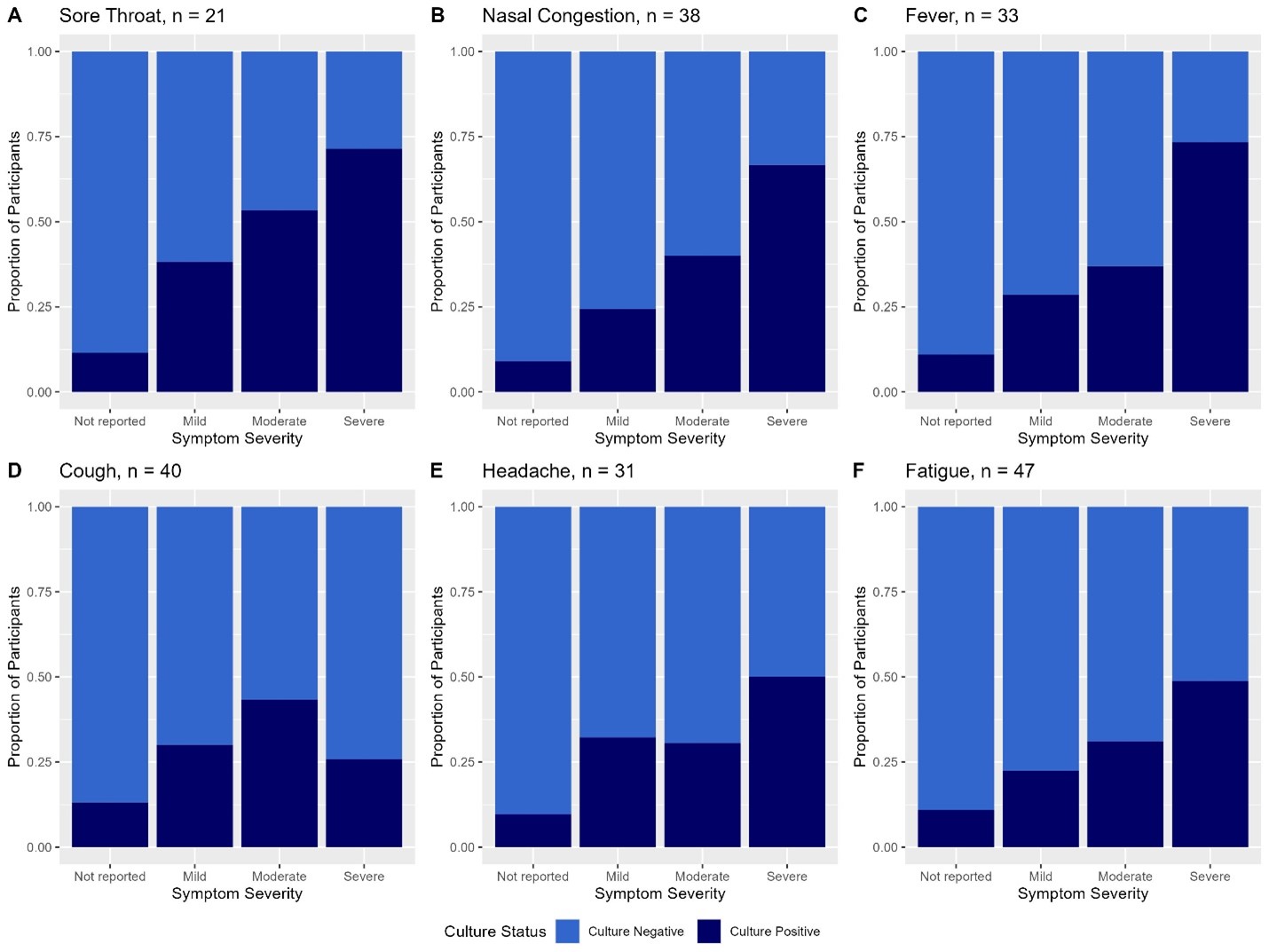

Supplement: Supplementary file 1 — Figure S1. Proportion of SARS‐CoV‐2 culture positive and culture negative specimens among symptomatic adults, by symptom severity—Nashville, TN, 2020. Symptom severity data comes from the validated Influenza Intensity and Impact Questionnaire (FluiiQ), which was asked of symptomatic participants aged ≥ 18 years. [file IRV-18-e13318-s001.jpg]
