# Supplementary material for: Association of Symptoms and Viral Culture Positivity for SARS‐CoV‐2—Tennessee, April–July 2020
Source: Influenza Other Respir Viruses. 2024 Jun 21;18(6):e13318. doi: 10.1111/irv.13318 (PMC11190945; doi:10.1111/irv.13318)
Supplement: Supplementary file 2 — Table S1. Odds of SARS‐CoV‐2 culture positivity among symptomatic individuals, by symptom—Nashville, TN, 2020: sensitivity analysis 1—exclusion of invalid specimens. Table S2. Odds of SARS‐CoV‐2 culture positivity among symptomatic individuals on PCR positive follow‐up days, by symptom—Nashville, TN, 2020: sensitivity analysis 2—exclusion of PCR negative results. Table S3. Odds of SARS‐CoV‐2 culture positivity among symptomatic individuals, by symptom and days since symptom onset—Nashville, TN, 2020. Table S4. Odds of SARS‐CoV‐2 culture positivity among symptomatic adults, by symptom severity—Nashville, TN, 2020. [file IRV-18-e13318-s002.docx]

**Supplemental Table A. Odds of SARS-CoV-2 culture positivity among symptomatic individuals, by symptom – Nashville, TN, 2020: Sensitivity Analysis 1 – Exclusion of Invalid Specimens**

|  | **Frequencies^1,2^** | | **Odds of Culture Positivity on Days with Symptom Compared to Days Without Symptom^1,2^** | | |
| --- | --- | --- | --- | --- | --- |
| **Symptom** | **Total Person-Days** | **Culture Positive Days / Days with Symptom** | **Model-based adjusted OR^3^** | **Model-based adjusted 95% CI^3^** | **Model-based**  **p-value^3^** |
| **Any Symptom^4^** | 705 | 154 / 518 (30%) | 1.94 | [0.99, 3.82] | 0.054 |
| **Wheezing** | 160 | 29 / 51 (57%) | 10.81 | [3.56, 32.83] | <0.0001 |
| **Runny Nose** | 391 | 74 / 145 (51%) | 3.49 | [1.91, 6.40] | <0.0001 |
| **Sore Throat** | 278 | 52 / 100 (52%) | 2.41 | [1.15, 5.04] | <0.05 |
| **Nasal Congestion** | 494 | 87 / 249 (35%) | 3.22 | [1.71, 6.06] | <0.0001 |
| **Fever** | 400 | 64 / 151 (42%) | 1.70 | [0.91, 3.19] | 0.098 |
| **Loss of Taste or Smell** | 451 | 65 / 255 (25%) | 3.29 | [1.37, 7.90] | <0.01 |
| **Shortness of Breath** | 302 | 42 / 126 (33%) | 1.82 | [0.89, 3.70] | 0.099 |
| **Headache** | 332 | 57 / 159 (36%) | 2.12 | [1.00, 4.49] | <0.05 |
| **Cough** | 507 | 104 / 294 (35%) | 1.40 | [0.83, 2.37] | 0.211 |
| **Chest Tightness or Pain** | 279 | 37 / 114 (32%) | 1.40 | [0.64, 3.05] | 0.398 |
| **Fatigue** | 599 | 108 / 339 (32%) | 1.10 | [0.66, 1.84] | 0.708 |

^1^Restricted to follow-up days after given symptom began. Each row is a separate model.

^2^Excluded specimens that were PCR invalid or inconclusive and specimens that were PCR positive with an unknown culture result

^3^An age-adjusted generalized additive model (GAM) with a binomial distribution using a logit link function was used to account for repeated measurements within participants, with a thin-plate spline to account for the non-linear relationship between symptom-day and culture status

^4^Any symptom is defined as having reported at least one symptom

**Supplemental Table B. Odds of SARS-CoV-2 culture positivity among symptomatic individuals on PCR positive follow-up days, by symptom – Nashville, TN, 2020: Sensitivity Analysis 2 – Exclusion of PCR Negative Results**

|  | **Frequencies^1,2^** | | **Odds of Culture Positivity on PCR Positive Days with Symptom Compared to PCR Positive Days Without Symptom^1,2^** | | |
| --- | --- | --- | --- | --- | --- |
| **Symptom** | **Total Person-Days** | **Culture Positive Days / Days with Symptom** | **Model-based adjusted OR^3^** | **Model-based adjusted 95% CI^3^** | **Model-based**  **p-value^3^** |
| **Any Symptom^4^** | 366 | 152 / 308 (49%) | 2.05 | [0.98, 4.30] | 0.06 |
| **Wheezing** | 74 | 28 / 39 (72%) | 6.29 | [1.68, 23.48] | <0.01 |
| **Runny Nose** | 216 | 73 / 103 (71%) | 3.79 | [1.94 7.42] | <0.0001 |
| **Sore Throat** | 150 | 51 / 81 (63%) | 2.39 | [1.05, 5.44] | <0.05 |
| **Nasal Congestion** | 236 | 85 / 159 (53%) | 2.32 | [1.15, 4.65] | <0.05 |
| **Fever** | 197 | 63 / 110 (57%) | 1.58 | [0.78, 3.18] | 0.20 |
| **Loss of Taste or Smell** | 196 | 64 / 150 (43%) | 3.82 | [1.38, 10.59] | <0.05 |
| **Shortness of Breath** | 128 | 41 / 79 (52%) | 1.49 | [0.66, 3.37] | 0.34 |
| **Headache** | 155 | 55 / 108 (51%) | 1.37 | [0.56, 3.34] | 0.49 |
| **Cough** | 276 | 102 / 185 (55%) | 1.55 | [0.88, 2.74] | 0.13 |
| **Chest Tightness or Pain** | 127 | 35 / 77 (45%) | 0.94 | [0.38, 2.32] | 0.89 |
| **Fatigue** | 303 | 106 / 212 (50%) | 0.82 | [0.46, 1.49] | 0.53 |

^1^Restricted to follow-up days after given symptom began. Each row is a separate model.

^2^Excluded specimens that were PCR negative

^3^An age-adjusted generalized additive model (GAM) with a binomial distribution using a logit link function was used to account for repeated measurements within participants, with a thin-plate spline to account for the non-linear relationship between symptom-day and culture status

^4^Any symptom is defined as having reported at least one symptom

**Supplemental Table C. Odds of SARS-CoV-2 culture positivity among symptomatic individuals, by symptom and days since symptom onset – Nashville, TN, 2020**

|  | **Odds of Culture Positivity on Days with Symptom Compared to Days Without Symptom^1^** | | | | | |
| --- | --- | --- | --- | --- | --- | --- |
|  | **Days 0-4 From Symptom Onset** | | | **Days 5+ From Symptom Onset** | | |
| **Symptom** | **Total Person-Days** | **Culture Positive Days / Days with Symptom** | **Crude OR** | **Total Person-Days** | **Culture Positive Days / Days with Symptom** | **Crude OR** |
| **Any Symptom^2^** | 292 | 129 / 262 (49%) | 3.69 | 456 | 25 / 288 (9%) | 1.62 |
| **Wheezing** | 73 | 25 / 36 (69%) | 2.85 | 98 | 4 / 17 (24%) | 9.53 |
| **Loss of Taste or Smell** | 202 | 59 / 164 (36%) | 4.56 | 278 | 6 / 107 (6%) | 1.92 |
| **Runny Nose** | 166 | 63 / 96 (66%) | 2.09 | 254 | 11 / 62 (18%) | 4.26 |
| **Nasal Congestion** | 204 | 77 / 147 (52%) | 2.71 | 316 | 10 / 116 (9%) | 2.87 |
| **Sore Throat** | 120 | 46 / 70 (66%) | 1.83 | 179 | 6 / 36 (17%) | 3.97 |
| **Fever** | 178 | 59 / 105 (56%) | 1.78 | 244 | 5 / 50 (10%) | 2.77 |
| **Headache** | 147 | 51 / 108 (47%) | 1.67 | 203 | 6 / 63 (10%) | 3.33 |
| **Shortness of Breath** | 141 | 40 / 89 (45%) | 2.60 | 186 | 2 / 44 (5%) | 0.72 |
| **Cough** | 209 | 86 / 158 (54%) | 1.54 | 332 | 18 / 148 (12%) | 1.72 |
| **Chest Tightness or Pain** | 126 | 33 / 81 (41%) | 1.31 | 176 | 4 / 40 (10%) | 2.72 |
| **Fatigue** | 254 | 94 / 192 (49%) | 1.45 | 385 | 14 / 174 (8%) | 1.62 |

^1^Restricted to follow-up days after given symptom began. Each row is a separate model.

^2^Any symptom is defined as having reported at least one symptom

**Supplemental Table D. Odds of SARS-CoV-2 culture positivity among symptomatic adults, by symptom severity – Nashville, TN, 2020**

| **Symptom** | **Severity** | **Number Culture Positives^1^** | **Number of Specimens^1^** | **Adjusted Odds Ratio^1,2^** | **95% CI** | **p value** |
| --- | --- | --- | --- | --- | --- | --- |
| **Sore Throat** | Not reported | 17 | 148 | Reference |  |  |
|  | Mild | 34 | 89 | 2.44 | [1.14, 5.26] | <0.05 |
|  | Moderate | 8 | 15 | 3.36 | [0.94, 12.05] | 0.06 |
|  | Severe | 5 | 7 | 8.30 | [1.21, 56.86] | <0.05 |
| **Nasal Congestion** | Not reported | 19 | 210 | Reference |  |  |
|  | Mild | 42 | 172 | 1.54 | [0.78, 3.03] | 0.21 |
|  | Moderate | 24 | 60 | 1.82 | [0.81, 4.08] | 0.15 |
|  | Severe | 8 | 12 | 15.16 | [3.72, 61.82] | <0.0001 |
| **Fever** | Not reported | 26 | 237 | Reference |  |  |
|  | Mild | 24 | 84 | 0.93 | [0.43, 2.03] | 0.86 |
|  | Moderate | 17 | 46 | 1.21 | [0.50, 2.94 | 0.67 |
|  | Severe | 11 | 15 | 12.46 | [3.31, 46.88] | <0.0001 |
| **Cough** | Not reported | 25 | 190 | Reference |  |  |
|  | Mild | 55 | 183 | 1.42 | [0.78, 2.60] | 0.25 |
|  | Moderate | 29 | 67 | 2.29 | [1.11, 4.70 | <0.05 |
|  | Severe | 8 | 31 | 1.38 | [0.53, 3.62] | 0.51 |
| **Headache** | Not reported | 19 | 196 | Reference |  |  |
|  | Mild | 40 | 124 | 1.97 | [0.96, 4.05] | 0.06 |
|  | Moderate | 15 | 49 | 1.83 | [0.74, 4.51] | 0.19 |
|  | Severe | 8 | 16 | 4.19 | [1.27, 13.85] | <0.05 |
| **Fatigue** | Not reported | 22 | 201 | Reference |  |  |
|  | Mild | 40 | 178 | 1.30 | [0.69, 2.45 | 0.41 |
|  | Moderate | 41 | 132 | 1.38 | [0.71, 2.68] | 0.34 |
|  | Severe | 20 | 41 | 2.99 | [1.29, 6.91] | <0.05 |

^1^Restricted to follow-up days after given symptom began

^2^An age-adjusted generalized additive model (GAM) with a binomial distribution using a logit link function was used to account for repeated measurements within participants, with a thin-plate spline to account for the non-linear relationship between symptom-day and culture status
